# Randomised Controlled Trial of Particles Used in Uterine fibRoid Embolisation (PURE): Non-Spherical Polyvinyl Alcohol Versus Calibrated Microspheres

**DOI:** 10.1007/s00270-021-02977-0

**Published:** 2022-01-04

**Authors:** Raj Das, Anita Wale, Seyed Ameli Renani, Lakshmi Ratnam, Leto Mailli, Joo-Young Chun, Sourav Das, Balpreet Duggal, Isaac Manyonda, Anna-Maria Belli

**Affiliations:** 1 Department of Radiology, St. George’s Hospital, St George’s Healthcare NHS Trust, London, SW17 0QT UK; 2 Department of Gynaecology, St. George’s Hospital, London, UK

**Keywords:** Embolisation, Fibroid, Leiomyoma, Embolic, Particle, Infarction

## Abstract

**Purpose:**

The PURE study is a randomised controlled trial (RCT) comparing the clinical and MRI outcomes of patients treated with non-spherical polyvinyl alcohol, ns-PVA (Contour PVA–Boston Scientific–355–500 & 500–700 microns) versus calibrated hydrogel microspheres (Embozene–Varian Inc–700 & 900 microns) for symptomatic uterine fibroids.

**Materials and Methods:**

Prospective, ethically approved non-sponsored RCT in 84 patients in a single UK tertiary IR unit, ISRCTN registry trial number ISRCTN18191539 in 2013 and 2014. All patients with symptomatic fibroid disease were eligible. UAE followed a standardised protocol with UFS-QOL and contrast-enhanced MRI before and 6 months post UAE. Outcome measures included: (1) Uterine Fibroid Symptom and Quality of Life questionnaire (UFS-QOL). (2) Percentage total and dominant fibroid infarction. (3) Uterine and dominant fibroid volume reduction. (4) Volume of embolics.

**Results:**

Sixty-three patients completed the QOL follow-up (33 ns-PVA vs 30 Embozenes), the groups were equivalent at baseline. Patients were followed up for 6 months following UAE. There was no significant difference in symptom scores or HR-QOL between ns-PVA and Embozenes, *p* = 0.67 and 0.21, respectively. 92.7% of patients treated with ns-PVA achieved > 90% dominant fibroid infarction versus 61.8% treated with Embozenes (*p* = 0.0016). 66% of patients treated with ns = PVA achieved > 90% total fibroid percentage infarction compared with 35% in the Embozene group (*p* = 0.011). The mean vials/syringes used were 5.2 with Embozenes versus 4.1 using PVA (*p* = 0.08).

**Conclusion:**

The PURE study informs IRs regarding the efficacy of embolic agents in UAE, with superior fibroid infarction on MRI using ns-PVA versus Embozenes however no significant difference in clinical outcomes at 6 months after UAE.

**Supplementary Information:**

The online version contains supplementary material available at 10.1007/s00270-021-02977-0.

## Introduction

The evidence base for the efficacy of uterine embolization (UAE) for the treatment of symptomatic fibroid disease has been firmly established and is based on robust and rigorous research [1–4] and registries [5]. The majority of these studies have used polyvinyl alcohol and Embosphere (Merit Medical) as the primary embolic agents, but over the years several newer embolic agents have become available. Tris-acryl gelatin microspheres (Embospheres, Merit Medical) have also been in use for UAE since 2000 and also comprise a large volume of experience. The newer agents include Acryl-amido PVA (Beadblock, Boston Scientific) and Polyzene-F coated hydrogel microspheres (Embozenes, Varian Medical Systems, previously Boston Scientific and CeloNova BioSciences). At present, there is little objective evidence to allow the interventionalist to choose between the “tried and tested” agents such as non-spherical PVA (ns-PVA) versus the “newer” calibrated microspheres such as Embozenes.

Tris-acryl gelatine microspheres (TAGM) are undoubtedly highly effective uterine embolic agents, these have been compared to ns-PVA in a number of randomised studies and most studies have yielded no significant difference with ns-PVA and TAGM both effective as embolic agents [6].

There have also been comparisons between spherical PVA/PVA microspheres and TAGM in a number of randomised studies which have found equivalence or superiority of TAGM over spherical PVA [7–10].

Whilst Embozenes are regarded in a similar category to Embospheres, comparative studies between Embozenes and other agents are limited. Smeets et al. [11] and Stampfl et al. [12] have shown good fibroid devascularisation and improvement in QOL, but clearly more studies are required to firmly establish their place.

A systematic review and meta-analysis and analysed 262 citations and included five randomised, controlled trials involving 295 women and five non-RCTs involving 617 women [13].The systematic review confirmed that the current evidence demonstrates superiority of Embospheres (TAGM) over spherical PVA but there were no reported differences in outcomes between any of the other agents. Overall, the study concluded that the comparison of embolic agents was limited by lack of randomised controlled trial data and further research is warranted.

In Europe and the UK, the greatest experience regarding embolic agents is with ns-PVA, which should therefore be regarded as the “gold standard” in any comparative research. The PURE study was therefore designed to compare the clinical and MRI outcomes of patients treated with non-spherical polyvinyl alcohol, ns-PVA (Contour PVA–Boston Scientific) versus calibrated hydrogel microspheres (Embozene–Varian Inc) for symptomatic uterine fibroids.

## Methods

### Study Design

The PURE (Particles used in Uterine fibRoid Embolisation) study is a prospective, single-centre, single-blinded randomised controlled trial comparing two commercially available embolisation agents namely: Contour non-spherical polyvinyl alcohol (ns-PVA; Boston Scientific, USA (initial sizing of 355–500 micron and subsequently upsized to 500–700 microns after 4 vials)) to Embozene Microspheres (calibrated hydrogel microspheres with polyzene coating; Varian Medical Systems (initial sizing of 700 microns, upsized to 900 microns). It was performed in a tertiary Interventional Radiology Unit in London, UK and received approval from the regional research ethics committee in October 2012. The trial was registered on an online registry: ISRCTN registry trial number ISRCTN18191539.

**Primary outcome measure** was quality of life (QoL) outcomes assessed with the validated Uterine Fibroid Symptom and Health-related Quality of Life questionnaire (**UFS-HRQOL**) before and 6 months after UAE. The questionnaire was completed by each patient pre-procedure (on the day of procedure) without influence and a further questionnaire was collated at the IR clinic appointment 6 months post UAE.

The **secondary outcome measure** was to compare fibroid infarction using contrast-enhanced MRI **(CE-MRI)** performed before and 6 months after UAE. The MRI was performed at the time of referral for UAE, approximately 2–3 months prior to procedure. The following MRI parameters were recorded:

1. Percentage fibroid infarction of total fibroid burden and dominant fibroid.
  These were both assessed on a gradient scale of: 100%; 90–99%, 75–90%; 50–74%; 25–50%; < 25% as per convention [15].
2. Uterine and dominant fibroid volume reductions.
  Three plane measurements were obtained in sagittal long axis, sagittal short axis and transverse plane.
  The uterine and dominant fibroid volumes were calculated via the prolate ellipse equation (long axis × short axis × transverse × 0.522) with percentage volume reductions calculated.

The primary outcome measure was the comparison of quality of life outcomes between the two groups. Based on the published literature no study had compared the difference in quality of life outcomes between PVA and Embozenes. Spies et al. [6] showed there was a change in mean quality of life score for patients treated with PVA of 23.1+/−23.4. In addition, Smeets et al. [11] reported quality of scores, from which the mean change in quality of life can be extrapolated of 29 points. Therefore, from this data a hypothesised difference of quality of life would be approximately six points. From our clinical experience we felt that the difference in scores would be greater than could be extrapolated from the published literature and therefore we embarked upon this hypothesis generating study to determine the potential difference in the QOL outcomes between the two groups and therefore formal sample size calculation was not performed. A sample size of 80 patients was chosen to allow for the loss of patients to follow-up with the intention that it would leave 60 patients for final analysis, 30 in each group.

### Patient Selection

Patients were recruited from IR and Myoma Clinics and criteria are described in Table 1. Serious adverse events, requiring or prolonging hospitalisation, or potentially life-threatening events were recorded and reported appropriately. Patients were recruited between 01.01.2013 and 31.03.2014 with active follow-up ending on 31.12.2014.

**Table 1:** Inclusion and exclusion criteria

Inclusion criteria	Exclusion criteria
All women between 18 and 55 years with symptomatic uterine fibroids Suitable for uterine artery embolisation	Participating in other clinical research trials Recent or ongoing pelvic inflammatory disease Severe radiographic contrast medium allergy Significant adenomyosis, as identified by transvaginal ultrasound or MRI. (Concurrent adenomyosis where fibroids are believed to the predominant cause of symptoms were considered to be eligible) Positive pregnancy test Refusal to accept hysterectomy, in the event of a perioperative complication Postmenopausal, as defined as greater than one year since previous menstrual period Suspected malignancy Age < 18 years Unable to provide informed consent due to incapacity (as defined by Mental Capacity Act 2005 or Adults with Incapacity (Scotland) Act 2000 A non-English speaker where translation or interpretation facilities were insufficient to guarantee informed consent

Block randomisation was performed in groups of 20 using computerised randomisation tables and the embolic agent to be used was revealed to the operator just prior to the procedure.

### Procedure and Technique

Participants underwent UAE in a standardised method with the only difference being the embolic agent between the two groups. The patients were blinded to the embolic agent; however, the operators and nurses were not blinded due to visual, technical and preparation differences between the agents. UAE procedures were performed by four Consultant Interventional Radiologists with 3 to > 20 years’ experience in a standardised fashion in the setting of a tertiary IR unit.

A bilateral femoral approach was used in all patients with ultrasound-guided arterial access and catheterisation of the uterine arteries using 4 Fr RIM catheters. 4 Fr RIM catheters (Cook Medical) were used via a cross-over technique to catheterise the uterine arteries using fluoroscopy or roadmap functions. In the majority of cases the 4Fr catheters were advanced to the transverse segments of the uterine arteries and microcatheters (Progreat 2.7 French (Terumo Interventional Systems) used only in cases of spasm or for cases with small uterine arteries or unfavourable anatomy. Ovarian arterial supply was noted and embolised when identified. Catheters were advanced to the transverse segments of the uterine arteries, or as far as required to ensure a stable position and free flow. Embolisation was performed according to the following protocols:

NsPVA–350–500 micron particles were used initially and if greater than four vials were used, then was upsized to 500–700 microns, then 900–1100 microns after a further four vials. In the case of Embozenes, embolisation was commenced with 700 microns and if greater than two syringes were used, then the particles were upsized to 900 microns. Embozenes 1100 microns were also used at the operator’s discretion.

With both embolic agents, the angiographic endpoint used was the stasis of flow to ten cardiac beats and avoidance of excessive reflux.

The volume of embolic agent required was recorded. Post-procedure manual compression was applied at the puncture sites and a patient-controlled analgesia (PCA) morphine pump and conventional a nalgesia and antibiotics were administered peri-operatively. All patients remained inpatients overnight for pain control.

Patient follow-up was as per the standard UAE protocol followed by our institution. This involved post-procedure information sheet, gynaecology clinic follow-up at 3 months post UAE, and MRI assessment and IR clinic follow-up at 6 months.

### Contrast-Enhanced MRI Technique

CE-MRI was performed routinely for all patients pre and 6 months post UAE on a GE 1.5 T MRI scanner with gadolinium enhanced pelvic MR angiography. The protocol included sagittal T2, Axial T1, T2, coronal STIR, axial T1 with fat suppression pre and post-contrast and coronal gadolinium enhanced MR angiography.

Two Consultant Radiologists, experienced in MRI fibroid interpretation with over 5 years’ experience, blinded to the embolic agent used, reviewed the MRI studies independently and any discrepancies were reviewed and interpreted by consensus.

### Data Analysis

Data were collected prospectively by a dedicated research fellow who maintained the study database. A clinical co-ordinator arranged appropriate follow-up and arranged for patients to receive the QOL life questionnaire. If patients did not respond they were contacted once further but due to limited funding further attempts to recall patients were not possible.

Statistical analysis was performed by SPSS 25.0.0 (SPSS, Chicago, IL.) on an intention-to-treat basis. *T* test and nonparametric 2-way comparison with Mann–Whitney U test was performed for the quality of life objective and differences between the groups in the infarction analysis was assessed using the a two-sided Fisher’s exact test. For all analyses, a *P*‐value of < 0.05 was considered significant. For comparison of the change in the percentage dominant fibroid infarction and total fibroid burden percentage infarction analysis was performed with patients grouped into those with > 90% change and < 90% as these cut-offs were deemed clinically relevant, and supported by previous studies [15, 16]. The CONSORT Statement for randomised controlled trials was completed for this study.

## Results

**Fig. 1:**
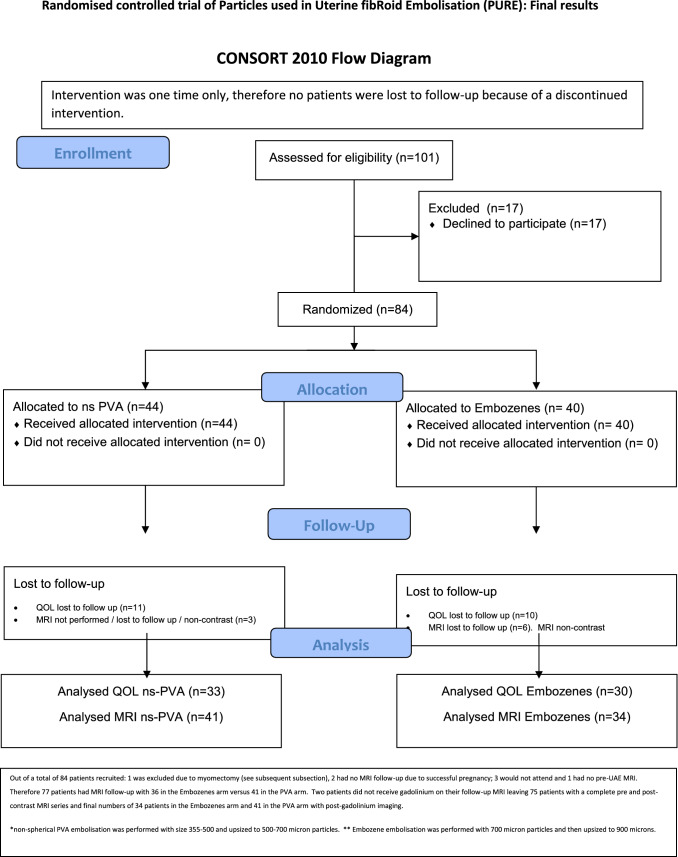
CONSORT (Consolidated standards of reporting trials) flow diagram

A total of 84 patients were recruited with a mean patient age of 41 years (Table 2). Technically successful bilateral UAE was performed in all patients. A total of 63 patients (75%) completed QOL follow-up to 6 months, 77 patients (92%) completed 6-month MRI follow-up enabling volume assessment, two of these patients did not received post-contrast imaging on the follow-up MRI and therefore 75 patients (89%) had contrast-enhanced MRI available for assessment of fibroid infarction as detailed in the CONSORT flow diagram (Fig. 1).

**Table 2:** Baseline characteristics of each group

		ns-PVA (*n* = 44)	Embozene (*n* = 40)
Age	Mean age	45.7	45.5
	Median age	45	45.5
	Range	30–56	30–56
Ethnicity	White British	9	4
	Asian	2	3
	Afro-Caribbean/black	24	15
	Other	8	18
Uterine and fibroid dimensions	Uterine volume (cm2)–pre-UAE	1046.3	952.3
	Dominant fibroid volume (cm2)–pre-UAE	304.5	246.6
QOL assessment	Fibroid-specific symptom score	56.9	60.2
	Fibroid-specific QOL total score	39.2	41.9

### Technical Factors

The mean volume of embolics in each group varied with a mean of 4.1 vials (range 2–9 vials) of PVA used and a mean 5.2 syringes (range = 1–12 syringe) with Embozenes, *p* = 0.08.

Ovarian arterial supply was noted and embolised when identified. (A unilateral ovarian arterial embolisation was required in 1 patient who was treated with Embozenes).

### Primary Outcome Measure: UFS-QOL and Symptom Score

The symptom scores and quality of life data were compared between the ns-PVA and Embozene arms. This demonstrated a pre-UAE symptom score of 55.9 in the ns-PVA group and 58.1 in the Embozene group and HR-QOL score of 39.1 in the ns-PVA group and 42.9 in the Embozene group, comparable at baseline.

Post UAE in the ns-PVA group, the symptom score was reduced to 26.6 and with a pre and post difference of 29.2 points (SD 26.58, 95% CI 19.8–38.63, *p* < 0.05). In the Embozene group, the symptom score was reduced to a mean of 32.8 with a difference of 25.3 points (SD 22.81, 95% CI 16.8–33.85, *p* < 0.05). Analysis of the pre and post difference therefore confirmed clinically effective UAE (*p* < 0.05) with respect to the effect of UAE on symptom score.

Despite a greater mean difference of 29.2 points in the ns-PVA group, compared to 25.3 points in the Embozene group there was no statistically significant difference in the mean change in symptom severity score between the two groups (*p* = 0.67), Table 3.

**Table 3:** Symptom severity, HR-QOL, uterine and dominant fibroid volumes–ns-PVA versus Embozenes

	ns-PVA (SD, 95% CI)	Embozene (SD, 95% CI)	*P* value
*Symptom Change (n* = 63)*	*n* = 33	*n* = 30	
Mean symptom severity score			
a. Pre-UAE	55.9	58.1	
b. Post UAE	26.6	32.8	
Mean change in symptom severity score	29.2 (26.6, 95% CI 19.8–38.6)	25.3 (22.8, 95% CI 16.8–33.9)	0.67
Mean HR quality of life (HR-QOL)			
a. Pre-UAE	39.1	42.9	
b. Post UAE	76.2	70.4	
Mean change in HR quality of life (HR-QOL)	37.2	27.5	0.21
*Follow-up imaging findings (n* = 75)**	*n* = 41	*n* = 34	
Mean uterine volume (cm^3^)			
a. Pre-UAE	10,463	9523	
b. Post UAE	6125	6477	
Mean change in uterine volume (%)	42	32	0.17
Mean dominant fibroid volume (cm^3^)			
a. Pre-UAE	3045	2466	
b. Post UAE	1548	1500	
Mean change in dominant fibroid volume (%)	50	39	0.56

Mann–Whitney U test

*63/84 patients completed both their pre- and post UAE symptom scores. **75/84 patients had pre- and post UAE MRI available for review

A similar relationship was demonstrated with respect to the HR-QOL difference, the QOL score increased in the ns-PVA group from 39.1 pre-UAE to 76.2 post UAE, with a QOL score difference of 37.2 (SD 30.5, 95% CI 26.4–47.9, *p* < 0.05). In the Embozene group the QOL score rose from 42.9 to 70.4 with a difference of 27.5 (SD 32.5, 95% CI 15.3–39.58, *p* < 0.05). Analysis of the pre and post difference therefore confirmed clinically effective UAE (*p* < 0.05) with respect to the effect of UAE on HR-QOL. Despite the greater mean difference of 37.2 points in the ns-PVA group, compared to 27.5 points in the Embozene group, there was no statistically significant difference between the ns-PVA and Embozenes groups (*p* = 0.21), Table 3.

### Secondary Outcome Measure: MRI Uterine and Dominant Fibroid Volume

A total of 77 patients of 84 recruited underwent MRI pre- and post UAE. Two patients were unable to receive gadolinium on the follow-up MRI and therefore 75 patients had pre- and post UAE post-contrast MRI available for assessment (Fig. 1).

The mean change in uterine volume post-embolisation was 28% in the ns—PVA group, versus 32% in the Embozene group (*p* = 0.51). The mean change in the dominant fibroid volume was 45% in the ns-PVA group versus 51% in the Embozene group, *p* = 0.17, Table 3.

### Secondary Outcome Measure: MRI Percentage Total Fibroid and Dominant Fibroid Infarction

A total of 75 patients had pre- and post UAE post-contrast MRI available for assessment with 41 patients in the ns-PVA group and 34 in the Embozene group.

92.7% of patients treated with ns-PVA achieved > 90% dominant fibroid infarction versus 61.8% treated with Embozenes (*p* = 0.0016). 66% of patients treated with ns-PVA achieved > 90% total fibroid percentage infarction compared with 35% in the Embozene group (*p* = 0.011), Table 4.

**Table 4:** Change in dominant fibroid volume and total fibroid burden between ns-PVA and Embozenes with a cut-off of > 90% versus < 90% infarction

	ns-PVA (%)	Embozene (%)	*P* value
	*n* = 41	*n* = 34	
*Percentage dominant fibroid infarction*			
> 90%	38 (92.7)	21 (61.8%)	0.0016
< 90%	3 (7.3)	13 (38.2%)	
*Total fibroid burden percentage infarction*			
> 90%	27 (66)	12 (35)	0.011
< 90%	14 (35)	22 (65)	

Fischer’s exact test

### Adverse Events

One patient experienced a major adverse event which was felt to be unrelated to the trial itself but an unexpected complication of uterine artery embolisation for a submucosal fibroid. This was considered a grade three complication according to CIRSE classification to report adverse events [14]. A 35-year-old patient underwent UAE with Embozenes for a solitary submucosal fibroid. Her initial recovery was uneventful but 10 days later she represented with vaginal bleeding, later requiring blood transfusion and MRI revealed uterine prolapse, then proceeding to laparotomy to correct uterine inversion and at that time myomectomy of the solitary fibroid was performed.

## Discussion

The PURE study aimed to compare quality of life outcomes and fibroid infarction on contrast-enhanced MRI following UAE with ns-PVA and Embozenes. The study showed there was a trend to greater improvement in HR-QOL in the ns-PVA arm, this was however not significant. In addition, the study showed that Embozenes resulted in significantly reduced total and dominant fibroid infarction when compared to ns-PVA, *p* = 0.011 and 0.0016, respectively. Prior studies have shown a clear correlation between incomplete fibroid infarction and clinical outcomes. Kroenke et al. [15] confirmed the finding that leiomyoma infarction above 90% on contrast-enhanced MR images after UAE correlated with significantly better symptom control and fewer reinterventions than in patients with a lower infarction rate. This was mirrored by a further study in 2012 by Scheurig-Muenkler [16] which also identified a correlation between treatment failure and < 90% fibroid devascularisation. However, these studies did not compare outcomes between ns-PVA and Embozenes, therefore the PURE study supports the 90% cut-off for clinically relevant infarction and consideration of the use of ns-PVA over Embozenes in this patient group.

There have also been other comparisons between embolic agents since the previous systematic review of 2014 [13]. A retrospective study comparison between nsPVA and Embospheres by Lacayo in 2017 [17] found no significant difference between these two groups and a further comparison between spherical PVA and Embospheres has also been performed with no significant difference reported [9]. Further RCT data comparing Gelfoam and tris-acryl gelatin microspheres (Embospheres) in the form of the RAGE trial [18] have been published as a pilot trial with relatively small numbers. To date there has been no comparative RCT involving Embozenes and ns-PVA.

Embozene has demonstrated a reduced leiomyoma infarction than ns-PVA in our study. There are a number of reasons as to why this may have occurred. One is a true difference in the embolisation capability of the two agents. The tightly calibrated particles of Embozene differ morphologically from the irregular non-spherical PVA particles that may clump together and cause more aggregation and vascular occlusion. In both arms of the study, the aim was to achieve stasis in the transverse segments of the uterine arteries. The two embolic agents were used to a similar endpoint (of angiographic stasis up to ten cardiac beats) and although Embozenes are postulated to require injection until sluggish forwards flow, rather than stasis, embolisation to the greater endpoint of complete stasis should confer a greater embolic effect. Finally, the sizes used differed slightly but according to standard clinical practice. Non-spherical PVA was used at 355–500 um for four vials then upsized to 500–700 um whereas Embozenes were used at 700 um and then upsized to 900 um and 1100 um.

The PURE Study recruited successfully and the study design allowed satisfactory consecutive recruitment with no major breaches or protocol revisions. Limitations included that the operator was aware of the embolic used, however due to the technical differences between the agents, this was a pragmatic approach and the embolic agents were used systematically in a similar fashion. All other parameters of the UAE procedure were standardised and performed by a small group of experienced IRs.

The PURE study found a slight difference in the number of vials of embolic agent used. This could potentially have a cost implication or cost effectiveness utility; however, since the difference was not statistically significant this was not felt to be within the remit of this study.

The trial was not funded externally and therefore there was no access to a trial nurse or research support which likely contributed to some of the loss to follow-up. A further limitation of the study was the relatively short-term follow-up of 6 months and 20% loss of patients to follow-up. Medium and longer term follow-up would have been desirable but unfortunately due to the PURE study being a non-sponsored, non-commercial trial, longer term follow-up could not be achieved and patients lost to follow-up could not be further identified. It is known that clinical failures from UAE can occur later than 6 months and therefore the full clinical impact of these findings cannot be fully reported. The prognostic value of MRI may also have a significance at over 12 months follow-up.

Use of the prolate ellipse method for evaluation of uterine volume has been used in several other UAE studies [16] and is in routine use within centres in the UK. Tracing software is being used by some centres to allow more accurate assessment of the multifibroid uterus, which rarely has a completely ovoid shape [19]. This technique was not available at our institution at the time of evaluation but may be a useful approach for the future. As all measurements in both cohorts were taken using the prolate ellipse method this is unlikely to have impacted on the findings.

In conclusion, the PURE study was a two-arm comparative RCT without commercial influence. Although the primary clinical endpoint of quality of life improvement did not demonstrate a significant difference, there was a significant difference in the degree of dominant and total fibroid infarction with a 90% cut-off. The findings support further investigation into the use of ns-PVA versus Embozenes with longer term follow-up to ascertain whether the decreased infarction rate with Embozenes translates into clinically relevant patient outcomes with longer follow-up and a larger cohort.

## Supplementary Information

Below is the link to the electronic supplementary material.

Supplementary file1 (DOCX 58 KB)
