# Supplementary material for: Randomised Controlled Trial of Particles Used in Uterine fibRoid Embolisation (PURE): Non-Spherical Polyvinyl Alcohol Versus Calibrated Microspheres
Source: Cardiovasc Intervent Radiol. 2022 Jan 4;45(2):207–15. doi: 10.1007/s00270-021-02977-0 (PMC8807446; doi:10.1007/s00270-021-02977-0)
Supplement: Supplementary file 1 — Supplementary file1 (DOCX 58 KB) [file 270_2021_2977_MOESM1_ESM.docx]

**Supplementary material**


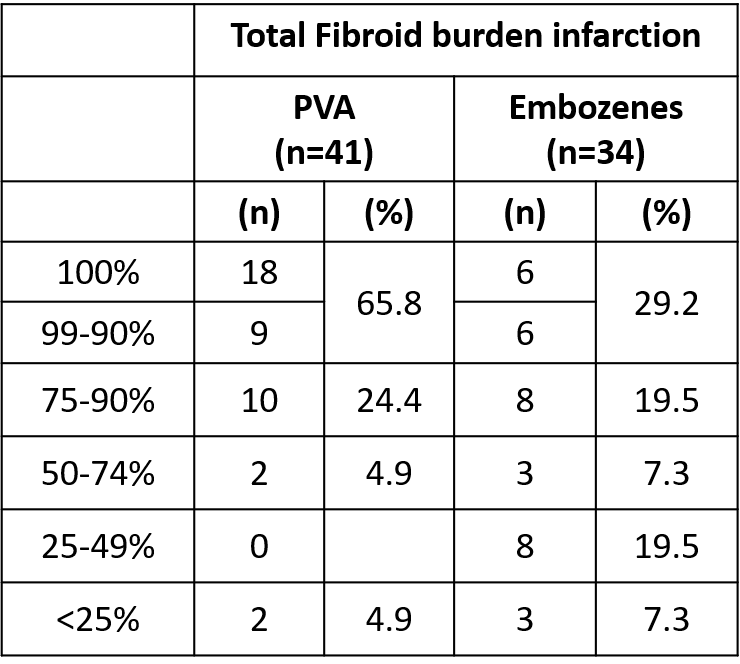

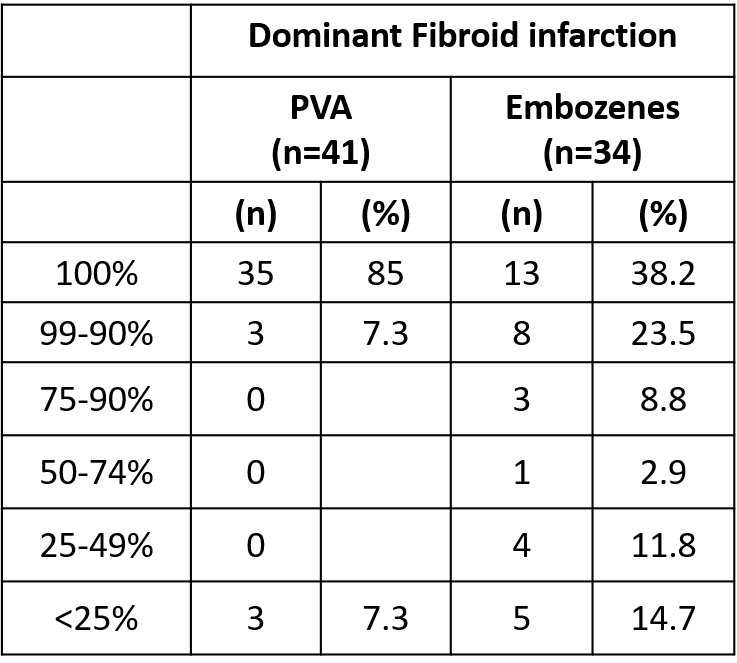


Table 5: Percentage dominant fibroid infarction and total fibroid burden infarction between PVA and Embozenes.

|  | Dominant Fibroid Infarction | | | |  | Total Fibroid burden infarction | | | |
| --- | --- | --- | --- | --- | --- | --- | --- | --- | --- |
|  | PVA (n=41) | | Embozenes (n=34) | |  | PVA (n=41) | | Embozenes (n=34) | |
|  | (n) | (%) | (n) | (%) |  | (n) | (%) | (n) | (%) |
| 100% | 35 | 85 | 13 | 38.2 | 100% | 18 | 65.8 | 6 | 29.2 |
| 90-99% | 3 | 7.3 | 8 | 23.5 | 90-99% | 9 |  | 6 |  |
| 75-90% | 0 |  | 3 | 8.8 | 75-90% | 10 | 24.4 | 8 | 19.5 |
| 50-74% | 0 |  | 1 | 2.9 | 50-74% | 2 | 4.9 | 3 | 7.3 |
| 25-49% | 0 |  | 4 | 11.8 | 25-49% | 0 |  | 8 | 19.5 |
| <25% | 3 | 7.3 | 5 | 14.7 | <25% | 2 | 4.9 | 3 | 7.3 |
|  | | | | | | | | | |
| Fischer’s Exact test | p < 0.0001 | | | |  | p= 0.02 | | | |

Table 6: Fischer’s exact test of dominant fibroid percentage infarction and total fibroid percentage infarction in both PVA and Embozenes groups.
